# The Advances in Research on the Pharmacological Effects of *Fructus Ligustri Lucidi*

**DOI:** 10.1155/2015/281873

**Published:** 2015-03-22

**Authors:** Zunting Pang, Zhou Zhi-yan, Wei Wang, Yanni Ma, Niu Feng-ju, Xuelan Zhang, Chunchao Han

**Affiliations:** ^1^School of Pharmacy, Shandong University of Traditional Chinese Medicine, Jinan 250355, China; ^2^School of Stomatology, Jilin University, Changchun 130021, China; ^3^Jinan Fifth People's Hospital, Jinan 250022, China

## Abstract

*Fructus Ligustri Lucidi* is a well-known invigorator in Chinese materia medica with hepatoprotective effect, anticancer activity, antioxidant activity, and so on. And oleanolic acids are the major pharmacologically active components in *Fructus Ligustri Lucidi*. So it has great value in medical health, and may be developed to a complementary and alternative medicine through further research. In this paper, the advances in research on pharmacological effects of *Fructus Ligustri Lucidi* were summarized by reviewing the recent related literature.

## 1. Introduction

*Fructus Ligustri Lucidi* (FLL, Nuzhenzi in Chinese) is the dried ripen fruit of* Ligustrum lucidum* Ait and it is a widely used herbal medicine for the prevention and treatment of a variety of pathologies. In the theory of Chinese traditional medicine, it has the effects of tonifying middle, calming five zang-organs, cultivating spirit, and nourishing the kidneys and liver. Besides, it can be applied to improve eyesight, replenish the liver and kidney, and promote the growth of black hair.

In recent decades, great progress about* Fructus Ligustri Lucidi* has been achieved by scholars inside and outside, and they have found a variety of active ingredients in* Fructus Ligustri Lucidi*, such as triterpenes, secoiridoid glucosides, volatile components, flavonoids, and phenolic compounds [1, 2]. In addition, modern pharmacological and chemical researches have indicated that* Fructus Ligustri Lucidi* has many pharmacological effects including hepatoprotective effect [3–5], anticancer activity [6, 7], antioxidant activity [8–10], immunomodulating effect [11], antiviral activity [12, 13], and antiosteoporosis activity [14–23] (Table 1). Although the clinical researches about pharmacological effects of* Fructus Ligustri Lucidi* are less,* Fructus Ligustri Lucidi* as the alternative chemotherapeutic and chemopreventive agents have recently received more and more attention.

## 2. The Pharmacological Effects of* Fructus Ligustri Lucidi*

### 2.1. Hepatoprotective Effect

**Figure 1:**
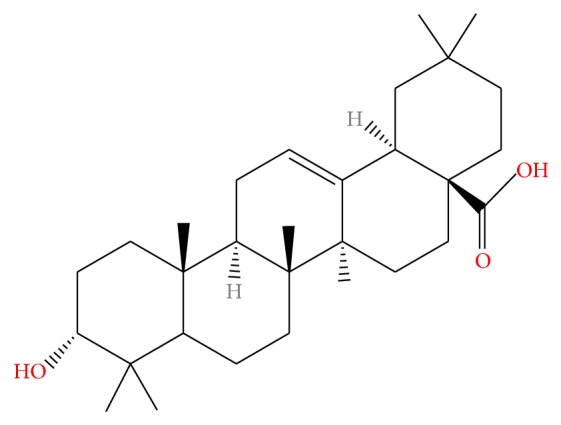
The structure of oleanolic acid (OA).

Oleanolic acid (OA, Figure 1) is a triterpenoid compound that exists widely in* Fructus Ligustri Lucidi*, and the hepatoprotective effect of OA was first reported in China and it has been used to treat liver disease in humans [3, 4]. Yim et al. have demonstrated that the fractions of chloroform and butanol derived from* Fructus Ligustri Lucidi*, which were enriched with oleanolic acid (OA), presented a dose-dependent protection against CCl_4_-induced hepatic injury* in vivo*. The promising hepatoprotective action may be associated with the enhancement of hepatic glutathione regeneration capacity (GRC), particularly under conditions of CCl_4_-induced oxidative stress [5].

### 2.2. Anticancer Activity

Liver cancer remains the fifth most common cancer in men and the seventh in women worldwide [24]. In China, traditional Chinese medicine (TCM) has a long history to treat liver cancer, and* Fructus Ligustri Lucidi* is known as the liver/kidney Yin tonifying herbs for liver cancer treatment [25]. Hu et al. have evaluated the effect of aqueous extracts of FFL on hepatocarcinoma cells, and significant apoptosis and cell senescence of hepatocellular carcinoma Bel-7402 cells by upregulating of p21 were observed [6].

On the other hand, because little information is available regarding the effect of FLL on glioma cell growth, Jeong et al. performed an experiment to investigate whether FLL extracts (extracted with methyl alcohol) affect glioma tumor growth. The results showed that glioma cell death could be caused by regulating the Akt/mTOR/survivin pathway* in vitro* [7].

### 2.3. Antioxidant Activity

Based on earlier reports, the human metabolic processes can produce harmful free radicals inevitably, which have been shown to possibly result in aging and other diseases [26, 27]. So, it is necessary to find effective radical scavengers in order to relieve the damaging effects of harmful free radicals.

**Figure 2:**
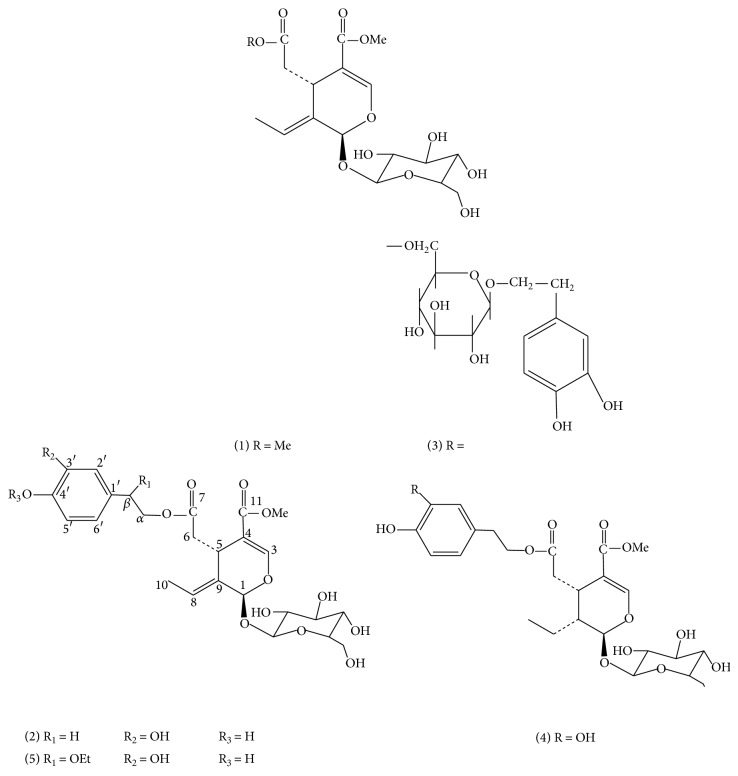
The structure of oleoside dimethyl ester, oleuropein, neonuezhenide, lucidumoside B, and lucidumoside C.

Zhen-Dan he et al. have isolated ten secoiridoid glucosides from FLL by bioassay-guided analysis, and their effects on free radical induced by hemolysis of RBC were tested. And five of them ((1) oleoside dimethyl ester, (2) oleuropein, (3) neonuezhenide, (4) lucidumoside B, and (5) lucidumoside C, Figure 2) showed significant inhibitory effects on the hemolysis of red blood cells induced by free radicals [8]. Ju et al. extracted FLL five times with 50% ethanol, and the crude extract was partitioned with four-times-volume amounts of n-butanol, chloroform, and ethyl acetate. Then they performed a series of antioxidant experiments* in vitro* to evaluate the antioxidant and protective properties of the different fractions against H_2_O_2_-induced oxidative damage in SH-SY5Y cells. They demonstrated that the phenolic-enriched ethyl acetate (EtOAc) fraction, whose major components are hydroxytyrosol and salidroside, was the most active part in scavenging free radicals and increasing the levels of antioxidant enzymes [9].

In addition, Lin et al. examined the antioxidant activities of ethanol extract of* Ligustrum lucidum fruits* (ELL) and its pharmacological effects on BHT-induced oxidative stress in rats. In their study, compared to the BHT-treated group (1000 mg/kg), the significant decrease in the levels of sGOT, sGPT, BUN, sALP, Cr, TG, LDH, BALF LDH, and lipid peroxides in liver and lung and the enhancement in the levels of antioxidant enzymes in these organs in BHT-treated rats were observed in the ELL-treated groups (250, 500, and 1000 mg/kg), which supported the protective effect of ELL against BHT-induced oxidative stress [10].

### 2.4. Immunomodulating Effect

As mentioned above, OA is the main effective constituent of FLL. Because OA can stimulate Th1 cells leading to the secretion of Th1 cytokines and then upregulates the Th1/Th2 arms resulting in raising the percentage of CD4^+^CD8^−^cells and promoting lymphocyte proliferation, OA has potential immunomodulatory effects. Wang et al. selected LLE as an immunoregulator, and they employed supercritical CO_2_ extraction technology to extract OA from FLL. Furthermore, immunomodulatory effect of OA on the immune cells of piglets was investigated through a series of experiments* in vitro*. Their results showed that OA, as an immunoadjuvant, has a beneficial and promising influence on the immune of piglets [11].

### 2.5. Antiviral Activity

**Figure 3:**
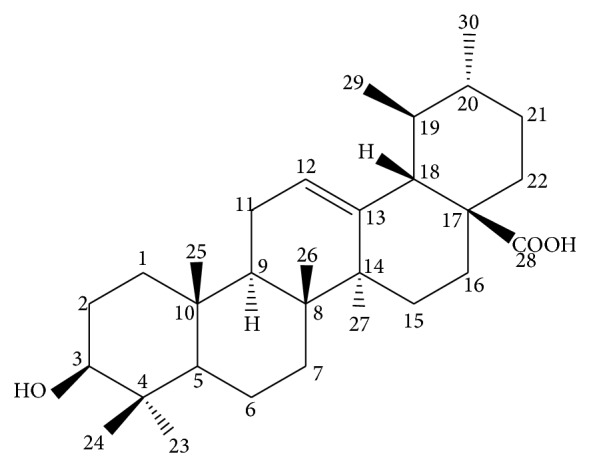
The structure of ursolic acid.

It is well-known that hepatitis C virus (HCV) infection is a serious worldwide problem, which causes significant mortality and morbidity. Some reports have showed that HCV affects about 3% of the global population and approximately 20–30% have developed liver disease, such as liver cirrhosis and chronic hepatitis. To date, current therapy with adverse side effects has curative powers in about 50% of patients infected with HCV genotype 1 and many chronically infected patients remain untreated [28–30]. So the progress of searching for effective antiviral drugs against HCV continues to be needed. And more and more researchers have studied the HCV inhibitors from FFL. Fortunately, accumulating evidence indicates that some active compounds of FLL were claimed to have potent antiviral activities against HCV. Lingbao Kong et al. found that oleanolic acid and ursolic acid (Figure 3) extracted from FLL are two antiviral components that possess anti-HCV activity based on their isomeric structures. They could suppress intracellular HCV NS5B RdRp activity. Moreover, they significantly inhibited the replication of HCV genotype 1b replicon and HCV genotype 2a JFH1 virus. By contrast, the results of their studies showed that oleanolic acid has one CH_3_ branched to last ring at C-20 position instead of C-19 position, so oleanolic acid had the better antiviral effect. In this way, the antiviral activity could be improved by modifying some compounds [12, 13].

### 2.6. Antiosteoporosis Activity

Fruit* Ligustrum lucidum* has long been used for the treatment of osteoporosis in China. Modern research in ovariectomized rats has demonstrated that the crude FLL extract could be useful to modulate the calcium balance and the turnover of bone [14–16]. Some reports showed that the ethanol extract of FLL (EFLL) could directly enhance the mineralization process resulting in improving bone properties and calcium balance in aged female rats [17–19]. These studies have provided evidence for the prevention and treatment of postmenopausal osteoporosis.

Ying Lyu et al. observed that FLL ethanol significantly increased bone mineral density and exerted beneficial improvement of bone mechanical properties in a dose-dependent manner by increasing Ca absorption and Ca retention, as well as reducing the RANKL/OPG ratio in growing female rats [20].

Estrogen deficiency and oxidative stress are considered as two major factors that cause the occurrence of osteoporosis [21, 22]. Thus, Chen et al. investigated the osteogenic constituents of FLL. They isolated and identified eight compounds from the aqueous extract of FLL (AFLL), namely, tyrosol (1), tyrosyl acetate (2), salidroside (3), hydroxytyrosol (4), oleoside dimethyl ester (5), nuzhenide (6), oleoside-7-ethyl-11-methyl ester (7), and G13 (8). Further studies showed that all eight compounds exhibited the antiosteoporotic effect with different mechanisms such as antioxidative effects and ER-dependent or independent pathways [23].

## 3. Conclusion

*Fructus Ligustri Lucidi* (FLL, Chinese name, Nuzhenzi) has been used in traditional Chinese medicine for over 1000 years. It contains a number of bioactive components. Moreover, FLL has been known to have hepatoprotective effect, anticancer activity, antioxidant activity, immunomodulating effect, antiviral activity, and antiosteoporosis activity, and other pharmacodynamic effects have been demonstrated as well, such as the treatment of coronary heart disease [31]. The chemical constituents of FLL include polysaccharides, triterpenes, secoiridoids, and flavonoids, which may be contributed to the pharmacological activities of FLL [32–34]. Therefore, the pharmacological effects and health function of* Fructus Ligustri Lucidi* are more and more focused on in the world.

## Figures and Tables

**Table 1:** The pharmacological effects and active compounds of *Fructus Ligustri Lucidi*.

The pharmacological effects	Active compounds	References
Hepatoprotective effect	Oleanolic acid	[3–5]
Anticancer activity	Aqueous extracts Methanol extracts	[6, 7]
Antioxidant activity	Secoiridoid glucosides Hydroxytyrosol and salidroside Ethanol extract	[8–10]
Immunomodulating effect	Oleanolic acid	[11]
Antiviral activity	Oleanolic acid Ursolic acid	[12, 13]
Antiosteoporosis activity	Ethanol extract Water extracts	[14–23]
